# Male–male associations in spotted bowerbirds (*Ptilonorhynchus maculatus*) exhibit attributes of courtship coalitions

**DOI:** 10.1007/s00265-022-03200-x

**Published:** 2022-07-07

**Authors:** Giovanni Spezie, Leonida Fusani

**Affiliations:** 1 Konrad Lorenz Institute of Ethology, University of Veterinary Medicine, Vienna, Austria; 2 Department of Behavioural and Cognitive Biology, University of Vienna, Vienna, Austria

**Keywords:** Courtship behaviour, Courtship learning, Coalitions, Same-sex associations, Ptilonorhynchidae

## Abstract

**Abstract:**

Despite strong selective pressures inherent in competition for mates, in species with non-resource-based mating systems males commonly engage in non-agonistic interactions with same-sex visitors at display arenas. Bowerbirds perform courtship dances on elaborate display structures — known as bowers — that are built and defended by one resident male. Several reports have suggested that bower owners tolerate the presence of specific male visitors at their display arenas, referred to here as ‘subordinates’. Subordinate males may learn the skills required for successful sexual signalling via prolonged social interactions at adults’ arenas, but little is known about whether courtship proficiency changes with experience and/or whether subordinates actively contribute to enhancing the resident male’s mating success. In this study, we investigated male-male associations in wild spotted bowerbirds (*Ptilonorhynchus maculatus*). We first sought to determine whether courtship behaviour differs based on bower ownership status. We then examined whether social interactions between bower owners and subordinate males may qualify as courtship coalitions. Our analysis of courtship postural components did not reveal differences in timing or relative occurrence of postural components between subordinate males and bower owners, whereas we found evidence that male-male associations in spotted bowerbirds may provide an example of rudimentary courtship coalitions. In particular, higher subordinate attendance is associated with lower destruction rates by neighbouring rivals and with overall higher mating success, and male pairs are stable in subsequent years. This study provides novel information about social dynamics among male bowerbirds, and further insights into the evolution of coalitionary behaviour in male displays.

**Significance statement:**

Same-sex associations between established males and subordinate visitors on display arenas are common in birds, yet poorly understood. Using video recordings from a population of wild spotted bowerbirds*, Ptilonorhynchus maculatus*, we performed a quantitative analysis on motor courtship components across males, and on their social interactions on display arenas to investigate the nature of male–male partnerships. Our results showed that motor courtship performance in subordinate visitors is not suggestive of an early ontogenetic stage, as previously speculated. Moreover, though bower ‘owners’ and subordinate males do not coordinate their behaviour during courtship or bower building, male–male partnerships may qualify as a rudimentary or incipient form of courtship coalitions. Subordinate males are tolerated at bowers, the magnitude of subordinate attendance correlates with owner males’ mating success, and repeated interactions between individuals reveal consistent partner associations.

**Supplementary Information:**

The online version contains supplementary material available at 10.1007/s00265-022-03200-x.

## Introduction

Competition among same-sex animals to attract and secure access to prospective mates is a tenet of sexual selection theory (Darwin 1871; Andersson 1994; Jones and Ratterman 2009; Kuijper et al. 2012). Variation in reproductive success caused by inter- and intra-sexual competition drives the evolution of secondary sexual traits and courtship displays (Jones and Ratterman 2009; McCullough et al. 2016; see also Lyon and Montgomerie 2012), as well as of different reproductive strategies (Emlen and Oring 1977; Bro-Jørgensen 2011; Shuster and Wade 2019). Among the variety of mating systems observed in nature, non-resource-based polygyny (hereafter NRP) represents a peculiar case of sexual competition, whereby males occupy and defend fixed display sites solely for the purpose of sexual signalling (Emlen and Oring 1977; Höglund and Alatalo 1995).

In the majority of NRP species, males display individually and compete with each other for attracting mates (Höglund and Alatalo 1995). However, despite strong selective pressures inherent in competing for fertilizations, social interactions among males on display arenas are not purely agonistic, and can fall into one of the following three categories. The first case is that of cooperatively displaying NRP birds and fish, in which multiple males coordinate courtship behaviour to collectively attract mates (reviewed in Díaz-Muñoz et al. 2014). For example, in manakins of the genus *Chiroxiphia*, one ‘alpha’ male synchronises courtship with multiple males on dedicated display perches and obtains all copulations (Foster 1977; McDonald 1989; DuVal 2007a, b). A second category is that of species without synchronised courtship, in which males nevertheless engage in complex social interactions with other courting neighbours. For example, male ruffs (*Calidris pugnax*) modulate aggression depending on the phenotype of the opponent and tolerate so-called ‘satellite’ males on their arena (van Rhijn 1983; Widemo 1998), and satin bowerbirds (*Ptilonorhynchus violaceus)* aggregate with kin and preferentially raid and destroy bowers of unrelated neighbours (Reynolds et al. 2009; but see Madden et al. 2004b). The third case is when resident males display individually, but tolerate and interact with sexually immature visitors that do not own a display court, referred to here as ‘subordinates’. For example, in some non-cooperatively displaying manakins, males are reported to display to and engage in social interactions with subordinate males of different age classes [white-fronted manakin (*Lepidothrix serena*) (Prum 1985; Théry 1990), whiteruffed manakin (*Corapipo altera*) (Jones et al. 2014), blue-crowned manakins (*Lepidothrix coronata*) (Durães 2009), golden-collared manakins (*Manacus vitellinus*) (Fusani and Schlinger 2012)]. During their visits, these males also perform courtship routines alone or with other subordinate males (Jones et al. 2014), yet ‘alpha’ males have control over subordinate activity, and these visitors are normally displaced when the ‘alpha’ males land on display arenas (e.g. Durães 2009). Similar interactions with so-called ‘auxiliary’ males (Isden 2014; Madden 2008) are commonly observed in bowerbirds (Ptilonorhynchidae) (Vellenga 1970, 1986; Maxwell 1999; Frith and Frith 2000a, b; Maxwell et al. 2004; Isden 2014) and birds-of-paradise (Paradisaeidae) (Frith and Cooper 1996; Frith et al. 1998). Despite the growing number of reports, data on subordinate visitors are scarce, particularly in NRP birds. Collecting systematic information on the behaviour of subordinate males is challenging, particularly in species where subordinate male plumage is morphologically indistinguishable from female plumage. Thus, while cooperatively displaying species have attracted much attention (e.g. Foster 1981; Ryder et al. 2009; DuVal 2013), relatively few studied have focused on the proximate and ultimate explanations for subordinate attendance.

The presence of subordinate males at established display arenas has been explained by some scholars as a form of apprenticeship (e.g. Madden 2008). Subordinate individuals may learn the skills required for mature sexual signalling via protracted social interactions at adults’ arenas. The idea that subordinate birds may gain such delayed benefits by associating with established males is referred to as the ‘skills hypothesis’ (Skutch 1961; Selander 1965; DuVal 2013). For example, in a study on satin bowerbirds *P. violaceus*, juveniles implanted with testosterone attained adult plumage prematurely, though implanted birds still showed poor coordination in bower-building and courtship behaviour (Collis and Borgia 1992, 1993). The authors concluded that the expression of mature sexual behaviour requires practice and sufficient exposure to appropriate social stimulation (Collis and Borgia 1993). To date, few studies have attempted to quantify socially mediated improvement in courtship competence, and the skills hypothesis has been investigated empirically in only one NRP model species to date (DuVal 2013, see also Trainer et al. 2002). A second possibility is that subordinate males may play an active role in contributing to resident males’ mating success, even in the absence of behavioural coordination during courtship. For instance, subordinate attendance at display arenas may directly or indirectly deter interferences from neighbouring competitors, their active presence may increase the conspicuousness of display arenas to females, or subordinate males may contribute to building and/or maintaining display arenas in species where sexual signalling involves modification of the environment (see Isden 2014).

Male bowerbirds construct a structure of sticks and straws (i.e. the bower), which is decorated with species-specific sets of colourful objects (Frith and Frith 2004). Bowers are fixed sites that are used by males to display an elaborate and multicomponent courtship routine to visiting females (Borgia 1995a, b; Madden 2002; Coleman et al. 2004; Frith and Frith 2004; Kelley and Endler 2012). Interactions with subordinate visitors appear to be a particularly complex phenomenon in this family (Madden 2008). In at least four species, established males regularly tolerate male visitors at their bowers (Frith and Frith 2000a, b; Maxwell et al. 2004; Madden 2008). In spotted bowerbirds (*Ptilonorhynchus maculatus*), for example, the same subordinate individuals have been seen at a bower repeatedly across breeding seasons (Isden 2014). These subordinate individuals appear to be mixed-age birds exhibiting immature plumage and/or incomplete and fragmentary courtship patterns (Madden 2008). There are similar reports in other bowerbird species of subordinate visitors engaging in multi-male displays (e.g. Vellenga 1970), which grants some plausibility to the practicing nature of these collective displays. Moreover, subordinate males have been anecdotally reported to participate in bower maintenance, although in an irregular and uncoordinated fashion (Vellenga 1970, 1986). Apart from these observational reports, there is no consensus regarding the benefits which may accrue to bower owners from establishing such male–male partnerships.

The aim of this study is to address this gap in the knowledge of male–male interactions in wild spotted bowerbirds *P. maculatus*. First, we test the skills hypothesis by conducting a systematic quantitative analysis of motor courtship performance in both subordinate males and bower owners. The skills hypothesis predicts that subordinate males should be less proficient in their courtship performance than long-term bower owners, based on the assumption that motor competence and courtship skills are perfected with time via protracted social interactions and practice. Although repeated observations have pointed to a possible role of motor learning (Vellenga 1986; Maxwell et al. 2004; Madden 2008), no previous study has quantified courtship proficiency in subordinate bowerbird males. Here, we compared courtship performance between bower owners and subordinate males, by exploring whether variation in timing and usage of different display moves depends on bower ownership status. In particular, we focus on a set of courtship parameters that relate to males’ responsiveness to receivers’ behaviour, in that they reflect how different display elements in their repertoire are used based on changes in the receiver’s spatial location. Prior research has shown that in order to produce attractive displays, male satin bowerbirds *P. violaceus* need to attend to audience reactions and adjust their motor performance accordingly (Patricelli et al. 2002, 2006). Here, we ask (i) whether bower owners in spotted bowerbirds also modulate courtship behaviour based on audience reactions and (ii) whether this ability varies with experience, i.e. bower owners are better able than subordinate males to flexibly adjust the type and timing of different display elements based on receiver behaviour.

Second, we investigate the nature of the interactions between bower owners and subordinate males. We ask in particular whether male associations in *P. maculatus* qualify as a form of coalitionary behaviour. Olson and Blumstein (2009) developed a definition based on three criteria for determining whether social interactions may be classified as coalitionary, which include: (1) mutual tolerance between coalition members, (2) collaboration against competitors and (3) preference for particular coalition partners during cooperative interactions. Using video recording data from a population of wild spotted bowerbirds, we examined whether (1) subordinate males engaged in male-specific behaviours at the bower in the presence of bower owners (tolerance); (2) the presence of subordinate males plays a role in increasing bower quality, overall mating success and/or in territory defence, in terms of reducing bower destructions by neighbouring competitors (collaboration); (3) subordinate males associate more often with particular bower owners (partner preference).

## Materials and methods

### Study site and subjects

We collected data from a population of wild spotted bowerbirds at Taunton National Park (Scientific), Queensland (23.54989S; 149.24088 E), during two breeding seasons (July–December 2018 and August–December 2019). Birds were mist-netted at bowers and marked with individual combinations of color bands. As subordinate males are morphologically indistinguishable from females (Madden et al. 2004a), blood samples were collected upon capture for genetic sexing (Supplementary Methods). Spotted bowerbirds can only be assigned to adult (2 +) or juvenile (first year) age categories based on morphological parameters (Higgins et al. 2006). Therefore, though the age of a few re-captured individuals could be approximated based on the date of first banding conducted during prior research on this population (Madden 2002, 2003; Madden et al. 2004a, b), detailed information about age was unknown for novel captures. In both 2018 and 2019, rainfall measurements at the field site were considerably below long-term averages (Bureau of Meteorology, Australian Government). In particular, during the 2019 breeding season bowers were gradually abandoned and remained virtually unattended towards the end of field activities (November–December 2019); no copulations were observed during field activities in 2019.

We monitored 14 active bowers using motion-activated camera traps (Browning Recon Force Advantage HD, 2018). Cameras were mounted on tripods and positioned in front of the bower (distance from camera, mean ± SD; 2018: 120 cm ± 43.20 cm; 2019: 177 cm ± 46.67 cm); video recordings were made at 30 frames per second. More details about camera recordings and calculations of activity budgets can be found in the Supplementary Methods.

### Display ethogram

**Fig. 1:**
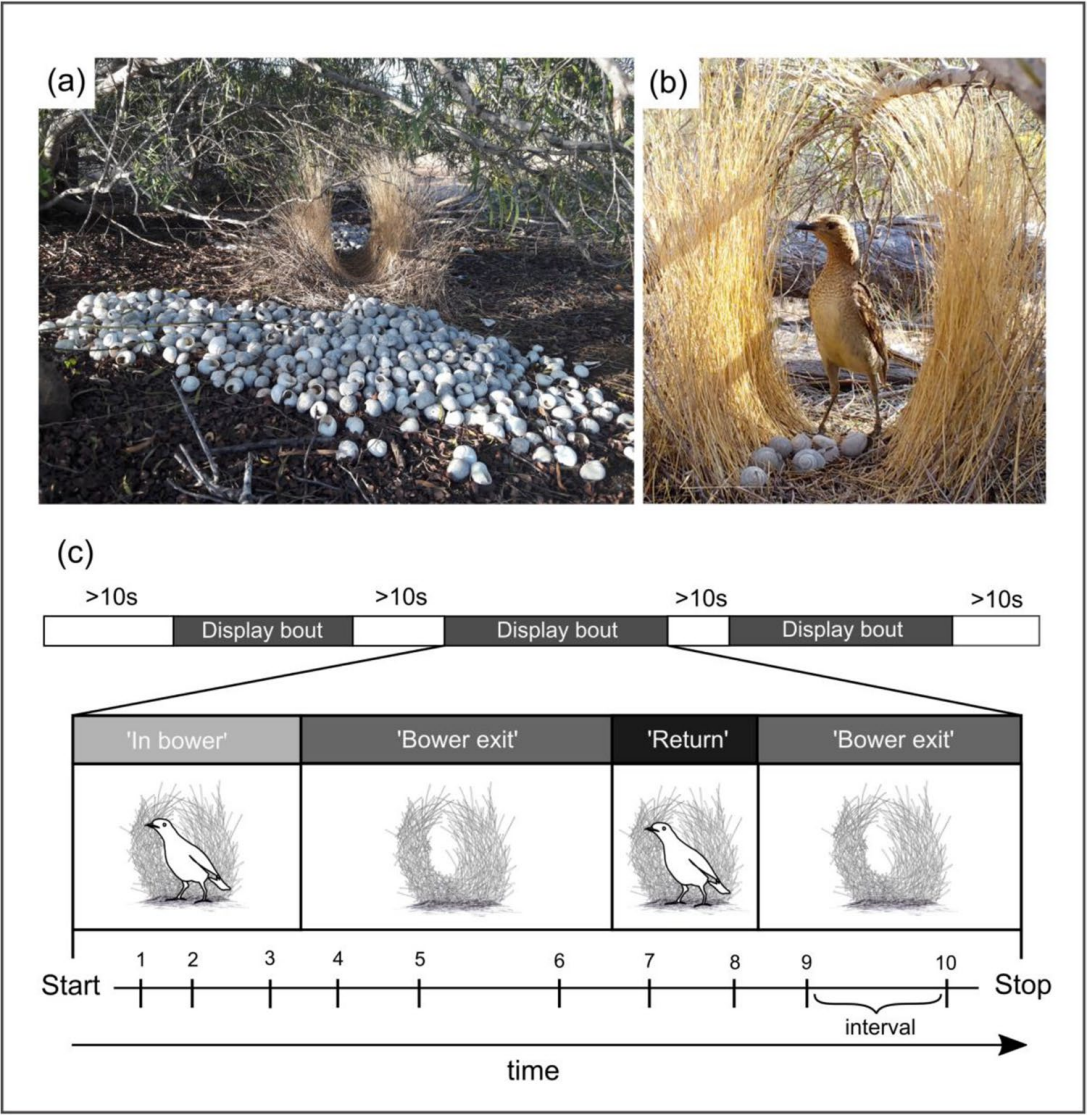
**a** Display arena of spotted bowerbird with pile of sun-bleached snail shells in the foreground and avenue-shaped bower in the background. Males arrange various decorations within the avenue walls and at the bower entrances. **b** A visitor positioning itself parallel to the bower walls while watching a displaying male. **c** Schematic depiction of a typical display sequence. Display bouts are defined here as courtship sequences separated by less than 10 s (top). We differentiate between different display segments depending on the position of the receiver during a display bout (middle). See text for definitions. During a display bout, males perform a repertoire of up to 19 stereotyped display elements (Supplementary Table S1) separated by intervals of variable length (bottom)

To determine the repertoire of motor display elements for courtship analysis, we scanned video recordings of courtship behaviour and compiled a descriptive ethogram (Supplementary Table S1). Display postures and movements in spotted bowerbirds were previously categorized as being either ‘peripheral’ or ‘central’, depending on the position of the courting male relative to the bower (Warham 1962; Frith and Frith 2004). During performance of peripheral displays, males typically move away from the bower and run in wide circles around the display arena (Frith and Frith 2004), whereas central elements are performed in proximity to the bower (Supplementary Video S1). We adopt here the above distinction and definition criteria for peripheral and central elements (Warham 1962), and we further differentiate within central elements between ‘static’ (standing) and ‘dynamic’ (moving towards the bower) display elements. While performing central dynamic elements males move towards the bower entrance with typical undulating movements (‘body ripple’; Supplementary Table S1), in some cases sprinting towards the female or violently slamming their body into the bower walls (‘mock attack’, Supplementary Video S2; Warham 1962; Borgia 1995b). Dynamic central elements thus involve high levels of vigour and normally cause the receiver to react by backing away or temporarily exiting the bower (Fig. 1c). Finally, we refer here to motionless pauses between subsequent display elements as ‘intervals’ (Fig. 1c).

### Analysis of courtship behaviour

#### Video scoring

We operationally define the start of a ‘courtship bout’ as the first display element performed when a visitor enters the bower walls (Fig. 1b, c). Intervals longer than 10 s or the departure of the courting male from the camera’s field of view were considered as the end of a courtship bout (Fig. 1c). To investigate whether courtship behaviour varied based on changes in receiver’s spatial location, we distinguished within courtship bouts between three segment types: ‘in bower’, ‘bower exit’ and ‘return’ segments (Fig. 1c). We define ‘in bower’ segments as the time between the first display element and a bower exit, i.e. the departure of the receiver from the space between the bower walls. A ‘bower exit’ segment is defined as the time between a bower exit and either the termination of the courtship bout, or the return of the receiver within the bower walls. This latter event, if present, marks the beginning of a ‘return’ segment (Fig. 1c).

After a preliminary screening of recorded videos, we restricted analysis to males with at least 600 s of video-recorded courtship behaviour. Males are classified as bower owners based on higher attendance at bowers, higher rates of bower maintenance and displaying (per hour of video recording), and lower rates of receiving displays from other males (see ‘Results’). Our final dataset included 22 males: 12 bower owners and 10 subordinate males. On average, 1887.18 ± 763.20 SD seconds of courtship behaviour per male (range: 718.03–3613.08 s) and 73.32 ± 25.74 SD courtship bouts per male (range: 24–118 bouts) were included in our analysis. Four raters who were blind to the aim of the study manually scored video recordings frame by frame based on our display ethogram using the software Loopy (http://loopb.io, Loopbio, GmbH, Austria). Inter-rater reliability calculated on a subset of 40 videos using the *kappa2* function of the *irr* R package (Gamer et al. 2019) showed strong agreement between raters (kappa = 0.857, *p* < 0.001).

#### Statistical analyses

For activity budgets, we used non-parametric two-samples Wilcoxon rank tests to compare rates of behaviours between males of different status categories, as dependent variables did not meet the assumption of normality.

For the analysis of courtship behaviour, we quantified from scored videos the following variables of visual displays: duration of display elements; proportion of peripheral, central static and central dynamic elements in each display segment category; length of intervals between subsequent display elements; and proportion of display elements performed with decorations held in the beak. First, we were interested in investigating whether these parameters of courtship behaviour vary across segments (in bower, bower exit, return). We hypothesized that male bowerbirds should respond to a change in receiver’s position by modifying courtship parameters, possibly to modulate threat levels. Second, we hypothesized that subordinate males differ from bower owners in responding to the behaviour of the receiver, i.e. in different display segments. To address these hypotheses, we investigated the effect of segment type, ownership status and their interaction on the above courtship parameters. The interaction term in our models was particularly important as we expected the effect of segment type to be more pronounced in bower owners than in subordinate males, if experience does play a role in shaping courtship performance.

For all models, we checked for collinearity issues among predictors using function *vif* (package *car*) to determine variance inflation factors, using 1 as threshold value (Quinn and Keough 2002; Field 2005). We included individual ID and courtship bout as random intercept effects, as we had repeated measures of different courtship parameters from the same males and from the same courtship bouts. As courtship was directed to unbanded birds, we could not consider audience ID as additional variable, though some displays may be repeated to the same individual (see Supplementary Methods). As a test of the overall effect of the predictors, all full models were compared to the respective null models comprising only the intercepts, random effects and control predictors (when present). Full-null model comparisons were performed using a likelihood ratio test (Dobson 2002). We assessed model stability by excluding the levels of the random effects one at a time (Nieuwenhuis et al. 2012). The function *emmeans* of the package *emmeans* (Lenth 2020) was used to perform post hoc tests. All analyses were performed in R 3.6.2 (R Core Team 2019).

- Display element durations
  To detect possible effects of the interaction between ownership status and display segment on the duration of eight display elements, we modelled durations with a Gaussian error distribution using GLMMs (generalized linear mixed models), running one separate model for each variable using the function *lmer* of the *lme4* package in R 3.6.2 (R Core Team 2019). We checked for the assumptions of normally distributed and homogeneous residuals by inspecting qqplots and scatterplots of residuals plotted against fitted values, which did not reveal deviations from these assumptions.

(b) Display element proportions
  To investigate whether bower owners and subordinate males differ in how they deploy display elements across courtship segments, we examined whether the proportion of peripheral, central static and central dynamic moves varied across segments based on ownership status. Continuous proportions (bound between 0 and 1) were modelled with a beta error distribution using the *glmmTMB* package (Brooks et al. 2017). Because low-intensity peripheral elements occurred more often after a bower exit (see ‘Results’), we additionally tested whether bower owners were faster than subordinate males in switching to peripheral elements. To assess this, we calculated the latency to switch to peripheral elements after a bower exit and compared latencies across males using a survival analysis approach, fitting a proportional hazards regression model with ownership status as predictor, using the *coxph* function of the *survival* R package (Therneau et al. 2021). In ‘bower exit’ segments where a peripheral element was not observed, the latency was treated as censored.
(c) Intervals
  We used number of frames as a count response variable to model the influence of segment type and ownership status on between-element interval duration. We compared a standard Poisson, a negative binomial, a zero-inflated Poisson and a zero-inflated negative binomial model based on AIC using the function *AICtab* of the package *bbmle*. We chose the negative binomial zero-inflated model based on its lowest AIC value. We included courtship segment type and ownership status as predictors both in the count part and in the zero part of the model, as both predictors may have an effect on the presence and length of the pauses the males take between subsequent display elements. We fitted the model using the function glmmTMB from the package *glmmTMB* (Brooks et al. 2017).
(d) Decoration use
  To investigate whether males differ in the way they use decorations during courtship, we compared the proportion of display elements that males displayed with a decoration in the beak between segments, display element category and ownership status. We modelled the proportions of display elements performed with a decoration held in the beak for all males with a beta error distribution, using the glmmTMB package (Brooks et al. 2017).

### Attributes of coalitionary behaviour

To test the hypothesis that male associations in *P. maculatus* exhibit one or more attributes of coalitionary behaviour (see ‘Introduction’), we extracted behavioural data from video recordings of male interactions at bowers.

- Mutual tolerance
  To quantify mutual tolerance among coalition members, we could not rely on aggressive interactions at bowers. Chases and physical aggressions were extremely rare, plausibly because these occur away from the arena and out of camera view, i.e. before undesired visitors land on the display ground (GS, personal observation). We therefore quantified tolerance as the time spent by subordinate males in male-specific behaviours (bower maintenance and courtship) in the presence of owners at their bower. For each bower, we compared the proportions of these male-specific behaviours to the proportions of other behaviours exhibited by subordinate males, i.e. the time spent alone at bowers, and the time spent as receivers or bystanders during displays of bower owners. Moreover, we were interested in investigating whether subordinate attendance was stable during the breeding season across bowers. We hypothesized that tolerance towards subordinate males would remain constant throughout the breeding season, if subordinate participation in bower activity contributes to enhancing overall attractiveness of a bower. We modelled the daily proportions of attendance (as time spent at the bower divided by total recording time per day per bower) of bower owners, subordinate males and unbanded individuals at each bower, using the *glmmTMB* package, specifying a beta error distribution (Brooks et al. 2017). Unbanded individuals may include females as well as subordinate males that we were unable to catch and mark (see above), though different assumptions about their identity is unlikely to affect the interpretation of our results, as we were interested in changes in proportions of attendance within a study period, and not differences in absolute proportions. Only days with uninterrupted camera activity were included into the analysis. Ownership status, date, and their interaction were set as fixed effects; we additionally included year as fixed effect, to check for overall differences in proportions of attendance between the two breeding seasons. To control for repeated measures per individual and recording day across bowers, we set these two variables as random effects in our model.
- Collaboration
  To quantify collaborative behaviours in subordinate males, we examined the relationship between subordinate attendance (i.e. averaged proportion of subordinate attendance per total video recording time per bower per year) and (i) destructions from neighbouring competitors, (ii) bower quality in terms of total number of decorations per bower and (iii) overall mating success of the bower owner (see Supplementary Methods for details). We predicted that higher subordinate attendance will show a positive relationship with number of displayed decorations and mating success, and a negative relationship with destruction rates from competitors. We ran separate linear models for the three count response variables (number of bower decorations, copulations and marauding events). Because the fitted Poisson models were clearly overdispersed (dispersion parameter > 1), we used a negative binomial error distribution, using the function *glm.nb* from the *MASS* package in R (Venables and Ripley 2002). Since sampling effort varied across bowers, we controlled for the effect of total recording time on marauding and mating events by including this variable as an offset term into the marauding and mating models (McCullagh and Nelder 1989).
- Partner preference
  To investigate coalition partner preference, we used a social network approach to determine whether male bowerbirds direct social interactions towards specific birds. As a measure of strength of association among male pairs, we calculated proximity events — i.e. the number of times two male birds were observed together — between each bower owner and subordinate males. We created separate social networks for each year using the package *igraph* in R. We calculated association strength based on a half-weight index. Association strengths calculated using an alternative simple-ratio index yield similar results; thus, only the results of the former index are shown here. To investigate whether subordinate males associate preferably with certain bower owners, we tested whether the observed networks differed from networks which were generated to simulate random associations among birds. These random networks aim to simulate a scenario where individuals interact equally often with all plausibly reachable bower owners in the study site. Random networks were generated using ‘pre-network’ permutations (Farine 2017); we permuted the observed data 10,000 times to construct surrogate networks that simulate the absence of social preference. To only allow for permutations that are ecologically realistic, we constrained permutations to occur only between birds observed at least once at a certain bower. By restricting swaps to within locations, we therefore controlled for a bird’s home range and excluded those social interactions which are impossible or unlikely to occur due to geographic distance between bower sites. We ran the permutation tests using the R packages *asnipe* (Farine 2013) and *sna* (Butts 2008) to calculate the network metric. Finally, to test whether preference patterns were consistent across years, we correlated the matrices representing the social networks for the two subsequent breeding seasons using a Mantel test in the R package *ape*, only for individuals and dyads observed in both years. The *p* values shown for the Mantel test are based on 10,000 permutations.

## Results

### Video recordings, display ethogram and activity budgets

**Fig. 2:**
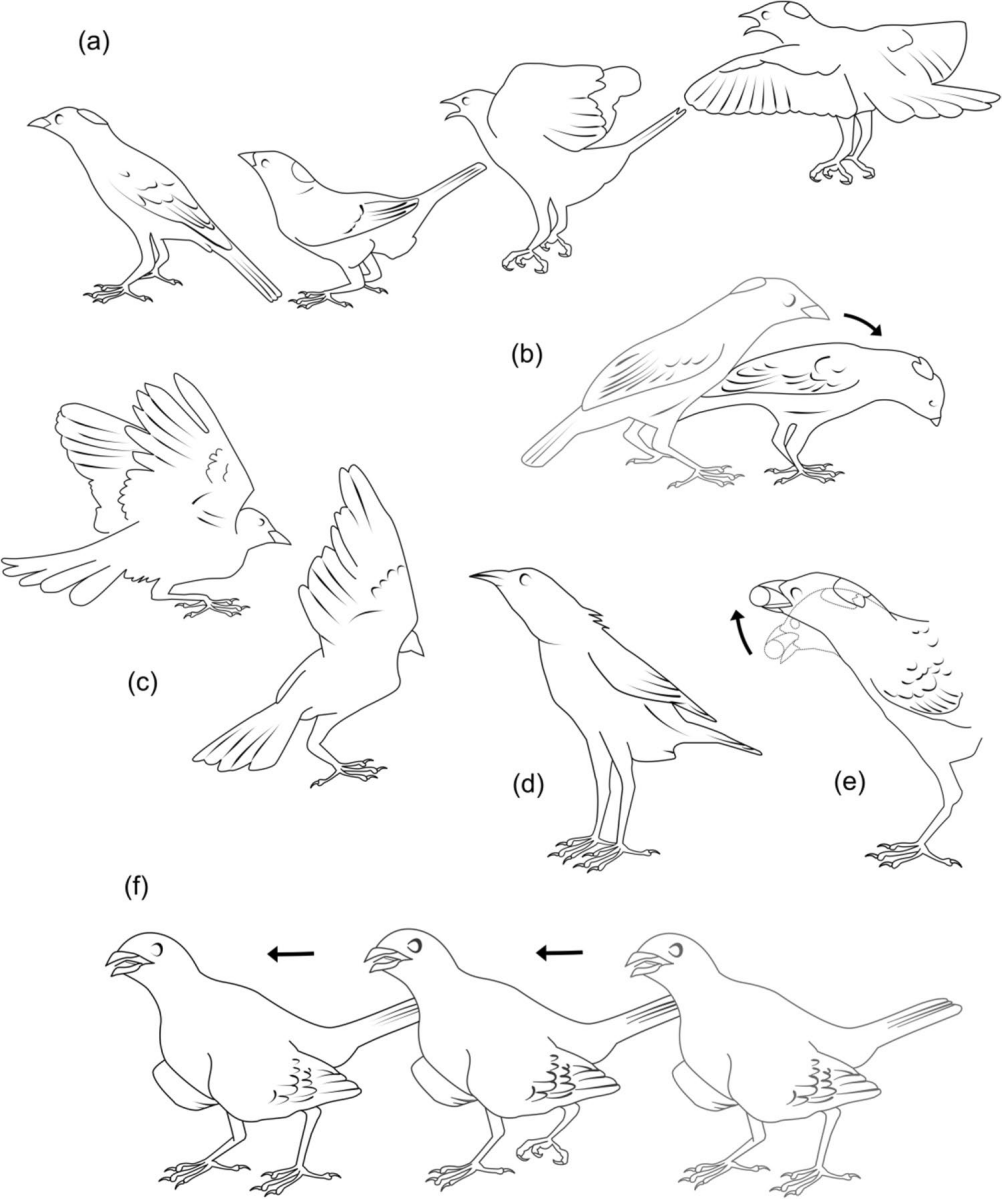
Six visual display elements exhibited by male spotted bowerbirds during courtship. **a** Leap. **b** Crest presentation. **c** Wing flick, in the two-wings variant (left) and single-wing variant (right). **d** Rising. **e** Nodding. **f** ‘Wings drooping’ strut. Other visual display elements are depicted in Warham (1962) and Frith and Frith (2004)

The sex of subordinate males was confirmed by genetic data (available for 77% of individuals in our sample). Total recording time and general activity budgets at bowers are summarized in Supplementary Table S2 and Fig. S1. Supplementary Table S3 and Fig. S2 provide descriptive statistics of social relationships at bowers in both breeding seasons. Bower owners exhibited higher rates of maintenance (*W* = 0, *p* < 0.001) and displaying behaviour (*W* = 13, *p* < 0.001), and lower rates of receiving displays from other birds (*W* = 218, *p* < 0.001) than subordinate males (Supplementary Fig. S3). We identified 19 different postural elements of male courtship (Fig. 2; Supplementary Table S1). We observed no changes in bower ownership between 2018 and 2019 across bowers, and only one ownership status change, with a male that was identified as subordinate in 2018 establishing a new bower site in 2019 (abandoned later in the season). We video recorded one copulation by an unbanded subordinate male in the absence of the owner, and three disruptions during copulations of bower owners by multiple unbanded and banded subordinate males (GS unpublished data).

### Analysis of courtship displays in bower owners and subordinate males

**Fig. 3:**
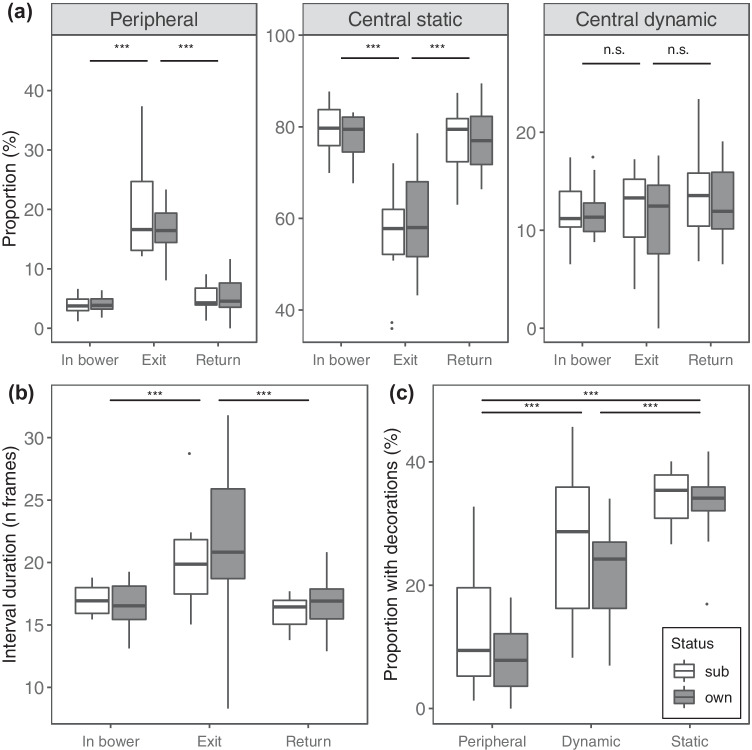
Comparisons between males with different ownership status (white = subordinate males; grey = bower owners) and in different display segments. **a** Comparison between the proportions of peripheral, central static and central dynamic display elements calculate for each male in different display segments; *N* = 66 observation, 22 birds. **b** Comparison between the durations of intervals calculated for each male in different display segments; *N* = 27,705 observations, 22 birds. **c** Comparison between proportions of display elements performed with a decoration held in the beak by each male; *N* = 138 observations, 22 birds. Box plots show median (black horizontal line), 25% and 75% quartiles, upper and lower values within 1.5 inter-quartile range (whiskers), and extreme values above 1.5 inter-quartile range (outliers); stars on top of horizontal bars depict significance levels: ***p < 0.001)
- Intervals
  Overall, the full model for interval length (in number of frames) fit significantly better than the null model lacking ownership status and segment type as predictors (full-null model comparison: *χ*^2^ = 234.36, *df* = 6, *P* < 0.001). More specifically, when the receiver left the bower, the intervals between subsequent display element were longer (Fig. 3b; Supplementary Table S6). Moreover, when the receiver left the bower, we found a higher probability of having ‘non-zero’ intervals between subsequent display elements (Supplementary Table S6). Thus, after a bower exit intervals were of longer duration and display elements were less likely to be performed in rapid succession. Again, males of different ownership status did not differ in average interval length (estimate ± SE =  − 0.01 ± 0.03; *z* =  − 0.28; *P* = 0.78) or in the probability of performing display elements separated by no interval (i.e. interval duration = 0) (estimate ± SE =  − 0.03 ± 0.03; *z* =  − 0.94; *P* = 0.35) (Supplementary Table S6).
- Decoration use
  The full model for proportions of display elements with decorations differed significantly from the null model (full-null model comparison: *χ*^2^ = 73.82, *df* = 3, *P* < 0.001). We found that peripheral elements were on average performed less often than central elements with a decoration held in the beak, and that central static elements had the highest average proportion of decoration use (Fig. 3c; Supplementary Table S7). Bower owners and subordinate males used decorations with similar proportions while performing display elements of the three categories (Supplementary Table S7).

- Display element durations
  We did not find an effect of ownership status and segment type on the duration of four display elements [head circling (*N* = 297): *χ*^2^ = 1.15, *df* = 3, *P* = 0.76; mock attack (*N* = 195): *χ*^2^ = 2.67, *df* = 3, *P* = 0.44; peripheral runs (*N* = 361): *χ*^2^ = 2.18, *df* = 3, *P* = 0.53; rising (*N* = 258): *χ*^2^ = 3.52, *df* = 3, *P* = 0.32]. The full models for duration of ‘body ripple’ and ‘crest presentation’ fit significantly better than their respective null models lacking ownership status and segment type as predictors [body ripple (*N* = 2280): *χ*^2^ = 9.52, *df* = 3, *P* = 0.02; crest presentation (*N* = 2656): *χ*^2^ = 10.70, *df* = 3, *P* = 0.01]. Segment type — but not ownership status — had an effect on element duration, with both ‘body ripple’ and ‘crest presentation’ being of significantly longer duration during ‘bower exit’ segments across males (Supplementary Table S4). Thus, the duration of all display elements under analysis did not differ between bower owners and subordinate males, and two display elements were overall of longer duration after receivers left the bower.
- Display element proportions
  The full models for proportions of peripheral (*χ*^2^ = 74.42, *df* = 5, *P* < 0.001) and central static (*χ*^2^ = 59.57, *df* = 5, *P* < 0.001) display elements fit significantly better than their respective null models lacking ownership status and segment type as predictors. In these two models, the interaction term was not significant; therefore, we fitted a reduced model lacking the interaction term to investigate the effect of the single fixed effects. We found that males responded to bower exits by increasing the proportion of peripheral elements and decreasing the proportions of central static elements (Fig. 3a; Supplementary Table S5). These differences were consistent across males irrespective of ownership status, as in both models ownership status did not have an effect on display element proportions (Supplementary Table S5). The model for proportions of central dynamic display elements did not fit significantly better than their respective null model lacking ownership status and segment type as predictors (*χ*^2^ = 5.53, *df* = 5, *P* = 0.35). We additionally predicted that the latency (in seconds) in switching to peripheral display elements after a bower exit differed based on ownership status (see ‘Methods’). The survival model showed that bower owners were not faster in responding to changes in receiver’s spatial location, as ownership status had no effect on latency (Supplementary Fig. S4).

### Attributes of coalitionary behaviour

#### Mutual tolerance

**Fig. 4:**
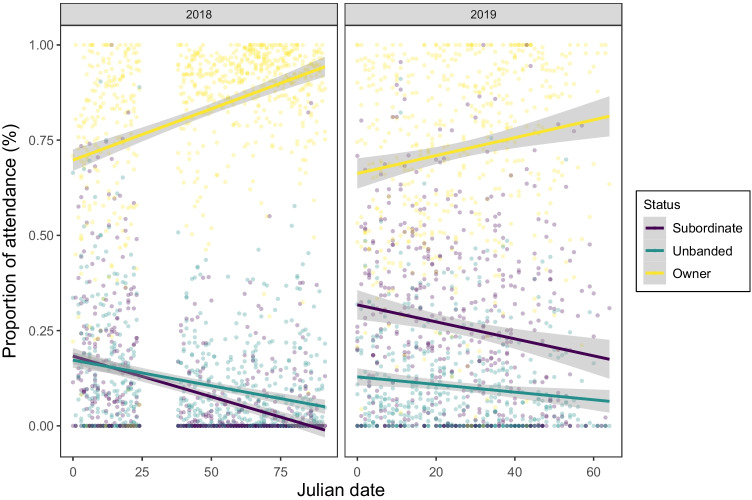
Proportions of attendance of bower owners, subordinate males and unbanded birds as a function of date. Proportions of attendance are total proportions of time per total video recording time per bower, calculated for each category of bird per day. Data are shown separately for 2018 and 2019. Regression lines are shown with 95% confidence intervals (grey shading). *N* = 3120 observations, 1226 days-bower

Subordinate males exhibited male-specific behaviours at the bowers in the presence of bower owners (displaying and bower building, both before and after marauding events), although they spent most of their time at bowers receiving or watching displays, or alone (Supplementary Fig. S5). Moreover, we found an overall effect of ownership status and date on the proportions of attendance at bowers (full-null model comparison: *χ*^2^ = 2817.6, *df* = 5, *P* < 0.001). More specifically, the effect of the interaction between ownership status and date was significant (*χ*^2^ = 339.58, *df* = 2, *P* < 0.001; Supplementary Table S8). In particular, subordinate males gradually decreased attendance at bowers as the mating season progressed in both years, whereas bower owners increased it (Fig. 4). Attendance was overall significantly higher during 2019 than 2018 (Supplementary Table S8).

#### Collaboration

**Fig. 5:**
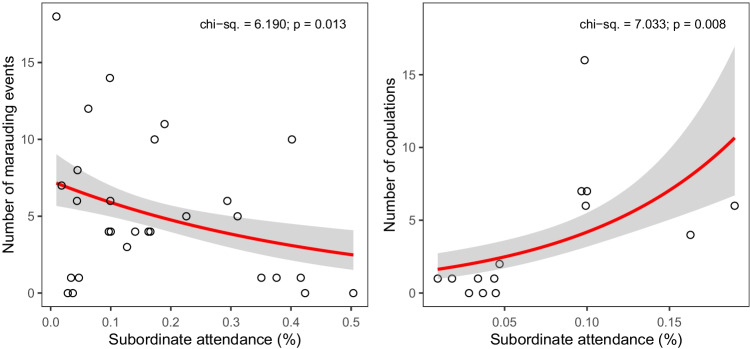
Effect of subordinate attendance on number of marauding events (left; *N* = 28 bowers-year) and on the number of copulations per bower (right; *N* = 14 bowers-year). Red lines depict the fitted model and grey areas depict 95% confidence intervals. Camera recording time is controlled for in both models by including this variable as an offset term (see ‘Methods’)

Subordinate attendance had a significant effect on the number of marauding events (*χ*^2^ = 6.190, *df* = 1, *P* = 0.013) and on the number of copulations (*χ*^2^ = 7.033, *df* = 1, *P* = 0.008). More specifically, bowers with higher subordinate attendance experienced fewer marauding events (estimate =  − 4.158, SE = 1.762, *z* =  − 2.360, *P* = 0.018) and more copulations during the 2018 breeding season (estimate = 14.555, SE = 4.586, *z* = 3.174, *P* = 0.002) (Fig. 5). However, the latter model showed poor stability, with estimates being highly variable after randomly removing data points. We did not find an effect of subordinate attendance on total number of displayed decorations (*χ*^2^ = 1.093, *df* = 1, *P* = 0.296) or number of snail shells (*χ*^2^ = 0.860, *df* = 1, *P* = 0.354).

#### Partner preference

The observed networks constructed with proximity events (Supplementary Fig. S6) differed from networks that were generated to simulate random associations among birds who were known to have been within possible home ranges. For both 2018 and 2019, the mean network metric (graph strength) was outside the 95% confidence interval of the simulated random networks (Supplementary Fig. S7), supporting the hypothesis of social preference and suggesting that subordinate bowerbirds associated more often than expected by chance with particular bower owners in both years. Moreover, the matrices representing the social networks were correlated between years (Mantel test: *Z*-statistic = 2.03; *P* = 0.001), indicating that male pairs are stable in consecutive years.

## Discussion

In this study, we tested two non-exclusive hypotheses for male–male associations in spotted bowerbirds. We first sought to determine whether subordinate males differ from bower owners based on a set of courtship parameters, including responsiveness to the receiver’s spatial location, inter-element interval duration, and spatial dependency of decoration use. We then investigated whether male-male social interactions in this species may qualify as courtship coalitions.

### Courtship parameters vary between display segments irrespective of ownership status

Our results show that male bowerbirds modify multiple parameters of their courtship routine depending on the position of the receiver, i.e. in different display segments defined by the departure of the receiver from the bower. These results suggest that spotted bowerbirds can respond rapidly to changes in receiver’s spatial location, and are in line with prior work on behavioural flexibility in bowerbirds. In satin bowerbirds *P. violaceus*, males adjust their motor performance based on audience reactions by modulating display intensity when receivers appear intolerant to the displayed aggression levels (Patricelli et al. 2002, 2006). Moreover, Borgia and Presgraves (1998) experimentally removed one of the bower walls, and showed that aggressive behaviours were more often displayed behind the standing wall, and not in front of the exposed receiver, again suggesting intensity modulation depending of bower configuration. Here, we show that male bowerbirds switch to peripheral elements, extend the intervals between single display elements, and increase the duration of specific display elements when the receiver leaves the display arena, suggesting a general decrease of display intensity. In particular, peripheral elements occur away from the bower walls and are likely associated with a decrease in the threat level perceived by the receiver. Yet, the frequency of highly vigorous ‘central dynamic’ elements was not affected by the position of the receiver.

Contrary to our prediction, however, subordinate males did not differ from bower owners in their responsiveness to the receiver’s spatial location or use of decorations during courtship. The data presented in this study argue against the skills hypothesis and indicate that alternative motor patterns may be available early in ontogeny or learned at an earlier stage during development (Spezie et al. 2022). These findings disagree with previous descriptive accounts on bowerbird courtship behaviour suggesting that birds with longer tenure at a bower show marked differences from other males which did not possess a permanent display arena. For instance, Vellenga (1970, 1986) reported an overall inability of subordinate males to perform mature-like courtship or bower building behaviour in satin bowerbirds *P. violaceus*. Doerr (2010) found that in great bowerbirds (*Ptilonorhynchus nuchalis*), males with more years of bower ownership exhibit lower rates of solitary displays, and concluded that more experienced individuals may not require practice of their courtship routine via individual motor training. Finally, it has been reported in a number of bowerbird species that subordinate males of different age classes gather in communal ‘practice’ arenas, where they perform fragmentary and immature versions of courtship routines as part of their development (Vellenga 1986; Madden 2008). Similar patterns of courtship development are observed in other NRP bird species. For instance, in swallow-tailed manakins (*Chiroxiphia caudata*), the so-called practice displays (i.e. courtship bouts performed by subordinate males) have been shown to differ from those of mature males (Ribeiro et al. 2019; Schaedler et al. 2021).

Our findings point instead to the possibility that subordinate males in this species may not associate with bower owners as part of a form of apprenticeship, but may rather obtain different benefits from establishing long-term partnerships. One hypothesis is that saturation of suitable display sites may force sexually mature subordinate males to “queue” in order to gain ownership of established arenas when these become available. Indeed, Isden (2014) showed that subordinate spotted bowerbirds are five times more likely to inherit the bower where they have served than other bowers. Moreover, the resulting male partnerships may allow subordinate males to establish dominance hierarchies with surrounding males and gain social competence (see below). Finally, the fact that subordinate males copulated or attempted to copulate in four separate instances supports the hypothesis that subordinate males may also obtain direct fitness benefits — i.e. occasional access to females – from attending established bowers.

A second possibility is that the age-dependent improvement in courtship competence was not captured by our analysis, due to the lack of precise information about the age of newly captured individuals (see ‘Methods’ section). This limitation would thus confound the results of a gross comparison between individuals of different ownership status. Male–male associations can last several years, and males require up to 7 years to establish a display arena and produce mature displays (Frith and Frith 2004). In this study, we could not observe subordinate males from the start of their association with bower owners and our dataset only covered two subsequent breeding seasons. Long-term longitudinal data about age and duration of male-male associations, as well as a focus on ‘practice’ arenas, would perhaps allow us to investigate the relationship between subordinate males’ age and courtship competence in more detail. Nevertheless, it is unlikely that subordinate males in our sample all belonged to the same age cohort and we therefore suggest that our results reflect an actual lack of differences in courtship behaviour between the two groups, at least for the parameters under analysis. It is also possible that the parameters we analysed may not be representative of differences in courtship skills among males. First, there could be variation in courtship behaviour directed towards females versus other immature males or alone (e.g., Barske et al. 2015), and these differences were not investigated in this study. Furthermore, prior work showed that female choice in some species operates on subtle or rapid movements, which may not be detected by human vision. For example, high-speed video recordings of courtship displays in golden-collared manakins *M. vitellinus* revealed that males show inter-individual variability in the courtship dance (Fusani et al. 2007) and that the speed with which specific courtship moves are executed by males can predict mating success (Barske et al. 2011). Thus, future studies should focus on fine-grained parameters of motor output, perhaps by means of automatic tracking technology for movement detection deploying machine learning algorithms (e.g. Valletta et al. 2017; Mathis et al. 2018, 2020; Janisch et al. 2021).

### Male–male associations in *P. maculatus* exhibit attributes of rudimentary coalitionary behaviour 

Our data provide evidence that subordinate males engage in male-specific behaviours — bower building and courtship — in the presence of bower owners across bowers, albeit at a lower rate than other behaviours such as receiving courtship. In addition, bower owners with higher subordinate attendance at their bowers suffer fewer destructions from neighbouring competitors and exhibit higher mating success. Finally, subordinate males direct their visits toward specific males within their home range, and these partnerships were stable across two consecutive breeding seasons. These results indicate that male-male associations in spotted bowerbirds meet the three criteria proposed by Olson and Blumstein (2009) for defining coalitionary behaviour: mutual tolerance, collaboration and partner preference.

We suggest that the described male–male associations in spotted bowerbirds provide evidence for rudimentary and/or incipient courtship coalitions. In their paper, Olson and Blumstein (2009) suggest describing courtship coalitions as a continuum, with complex and obligate forms of coordinated behaviour being located at one end of the spectrum, and mutual tolerance at the other (see also Díaz-Muñoz et al. 2014). Indeed, the degree to which collectively displaying animals coordinate behaviour to attract mates shows marked variation across species. Manakins (fam. Pipridae) exhibit variability in the degree of behavioural coordination, also within the same species. For instance, male white-ruffed manakins *C. altera* and white-fronted manakins (*Pipra serena*) facultatively participate in cooperative multi-male displays, while a proportion of breeding males nonetheless gets access to mates by displaying singly (Théry 1990; Jones et al. 2014). In wild turkeys (*Meleagris gallopavo*), cooperative males establish competitive coalitions on display arenas, which help them monopolize and defend access to females (Krakauer 2005; Krakauer and DuVal 2012). In this species, approximately half of the remaining males on the lek does not form coalitions (Krakauer and DuVal 2012). Finally, cooperative behaviour in the context of mate attraction often consists of mere mutual tolerance, whereby behavioural coordination is limited to the fact that males refrain from attacking coalition partners but share the benefits of displaying in physical proximity [bottlenose dolphins establish (Connor et al. 1992); wild horses (Feh 1999); cheetahs (Caro 1994)]. Male–male association in bowerbirds may thus represent a novel instance of coalitionary courtship partnerships, where both coalition partners benefit from reduced aggression towards specific individuals.

Nonetheless, we emphasise that subordinate males rarely exhibited courtship behaviour in the presence of bower owners, and we found no evidence of active contribution to bower building/maintenance by subordinates, for example in terms of the number of displayed decorations at bowers. Thus, the evidence for behavioural coordination in sexual signalling is limited to mutual tolerance and non-simultaneous courtship displays at the same bower. Moreover, several aspects of the described male associations in bowerbirds remain unclear. First, bower owners should tolerate subordinate presence at their bowers throughout the breeding season, if subordinate attendance plays a role in securing access to mates. By contrast, our results show that subordinate attendance decreased during the mating season across bowers, and was particularly low when females were receptive. One possibility is that while bower owners may benefit from subordinate presence early in the breeding season, additional costs — such as interference during mating — may offset the benefits of tolerating their presence when copulations start. Alternatively, same-sex interactions at the bowers such as multi-male displays may serve to establish dominance hierarchies prior to the onset of the breeding season. For example, in *P. serena*, a non-cooperatively displaying manakin species, coordinated displays between resident and subordinate males have been defined as being “more competitive than cooperative” (Prum 1985; see also Théry 1990). It has also been suggested that competitive interactions and aggressive displays may have been co-opted in cooperative contexts (Krakauer and DuVal 2012).

Second, the relationships we found between subordinate attendance, bower destructions from competitors and mating success are correlational and do not necessarily imply causation. Instead, it is possible that subordinate attendance may be a consequence — rather than a cause — of higher mating success and territory defence. For example, subordinate males may preferably choose as coalition partners high-quality males that are successful at repelling competitors. We could not quantify the degree to which subordinate males actively contributed to defending display arenas from neighbouring males’ raids, as the field of view of our camera traps was limited to a few square meters around the display arena. Nevertheless, it is plausible that the presence of any displaying or maintaining bird on a bower can discourage competing bower owners from marauding, as well as attract and/or affect females’ mating decisions, though this hypothesis requires further experimental support, e.g. by simulating subordinate activity via playback experiments. Tolerance towards subordinate males may thus benefit bower owners via a mere increase in the overall activity at their bowers, thus indirectly enhancing their attractiveness. In ruffs *C. pugnax*, for instance, resident males with high frequencies of visits by satellite males are more successful at mating (Hill 1991). Future studies should collect more detailed information about bower defence from subordinate males to shed light on the link between subordinate attendance and bower attractiveness.

Finally, information on the precise mechanism of partner preference is still lacking. How are these coalitions formed? Do subordinate males selectively choose their model, or do bower owners tolerate some subordinate males and repel others? It may be the case that male associations are explained by inclusive fitness benefits which accrue to bower owners from tolerating related subordinate males. Increased inclusive fitness benefits have been invoked to explain courtship coalitions in those species where partnerships are formed with kin. In turkeys *M. gallopavo*, subordinate coalition members forego access to mating, but obtain higher fitness values by collaborating with genetically related coalition partners rather than by displaying alone (Krakauer 2005). Future work should focus on kin relationships between bower owners and their subordinates to investigate possible benefits deriving from relatedness and kin selection.

## Supplementary Information

Below is the link to the electronic supplementary material.

Supplementary file1 (MP4 49688 KB)

Supplementary file2 (MP4 5763 KB)

Supplementary file3 (DOCX 156 KB)

## Data Availability

All datasets generated and analysed in this study may be accessed here: https://phaidra.vetmeduni.ac.at/o:1007.
