# Supplementary material for: Male–male associations in spotted bowerbirds (Ptilonorhynchus maculatus) exhibit attributes of courtship coalitions
Source: Behav Ecol Sociobiol. 2022 Jul 7;76(7):97. doi: 10.1007/s00265-022-03200-x (PMC9262789; doi:10.1007/s00265-022-03200-x)
Supplement: Supplementary file 3 — Supplementary file3 (DOCX 156 KB) [file 265_2022_3200_MOESM3_ESM.docx]

**Supplementary Material**

**Male-male associations in spotted bowerbirds (*Ptilonorhynchus maculatus*) exhibit attributes of courtship coalitions**

Giovanni Spezie^1,*^ and Leonida Fusani^1,2^

*^1^ Konrad Lorenz Institute of Ethology, University of Veterinary Medicine, Vienna, Austria*

*^2^ Department of Behavioural and Cognitive Biology, University of Vienna, Vienna, Austria*

Journal: *Behavioral Ecology and Sociobiology*

**Supplementary Methods**

*Study subjects, video recordings, activity budgets and bower measurements*

Birds were genetically sexed by PCR amplification, using P2 and P8 primers (Griffiths et al. 1998). DNA fragmentation was not needed prior to amplification and amplified DNA was run on agarose gel; samples from female birds showed two bands, those from males a single band. Information of genetic sexing is available for approximately 77% of the individuals that were used for analysis. All the individuals exhibiting male-specific behaviours turned out to be males after genetic sexing, therefore it is very likely that all other non-tested individuals that exhibited male-specific behaviours were also males.

Courtship displays in *P. maculatus* vary in duration from a few minutes up to 80 minutes. Camera traps were set to record videos continuously for as long as motion was detected. When activity at bowers lasted longer than maximum recording duration (5 minutes), there was a gap in footage of approximately 1s between subsequent videos (trigger and recovery speed were set to 0.4 s and 0.6 s, respectively). Male bowerbirds most commonly display to a single (male or female) receiver, but solitary displays (i.e. directed to no audience, or audience outside camera shot) as well as displays to multiple visitors are frequent. Displays to multiple visitors are chaotic, such that courtship performance is frequently disrupted by interference from bystanders and chases among individuals (GS, personal observation). To control for variation in quality of camera recordings and number of display receivers, we restricted our analysis to courtship interactions which fulfilled the following criteria: (1) minimum duration of two minutes; (2) courtship directed to a single receiver; (3) full visibility of the displaying male (i.e. not hidden by bower walls or vegetation). Due to the impossibility of catching and marking all individuals within the study population, video recordings at bowers include footage of unbanded birds of unknown identity and sex. Analyses of courtship behaviour by bower owners and subordinate males (and their interactions at bowers) were restricted to banded individuals. However, in video recordings of courtship behaviour the majority of visiting females was not banded and we could not be sure that the receiver was indeed a female, and not an immature male. We do show in this study that subordinate males commonly observe courtship by other males from inside the bower, and immature males are morphologically indistinguishable from females (see main text). In the calculations of activity budgets, unbanded birds are scored as a separate category.

Activity budgets were calculated as proportions of each particular behaviour per total video recording time per bower: (i)‘bower attendance’ is the time spent by a particular individual at a bower, irrespective of the specific activity observed; (ii) ‘bower maintenance’ refers to an individual constructing the bower, or replacing and rearranging decorations on the display arena; (iii) ‘display to other’ is the time spent by an individual performing courtship to a male or female conspecific bird; (iv) ‘solo display’ is the time spent by an individual performing courtship to no (visible) audience; (v) ‘receiving display’ is the time spent by an individual receiving courtship from another bird, normally from inside the bower walls.

Copulations were counted as discrete events identifiable by the displaying male’s contact with a female after the female solicited to the male by crouching inside the bower walls. When the identity of mating females was unknown, subsequent copulations spaced less than 1 hour from each other were counted as a single copulation event, as banded females were observed visiting and copulating repeatedly with the same male within short intervals (GS, personal observation; see also Borgia 1995a). Bower destructions (referred to as ‘marauding’; Madden et al. 2004b) by neighbouring males were also counted as discrete events and were clearly identifiable as violent tugging of the bower walls, scattering of the bower material and stealing of bower decorations (i.e. bowerbird collecting a decoration and departing from the display arena). As above, repeated bower destructions and decoration thefts within one hour by the same raiding neighbouring male were scored as single marauding events.

Spotted bowerbirds collect and arrange various types of decoration (green berries, red seed pods, glass, etc.) inside and in proximity of the bower avenue (Borgia 1995a; Madden 2002, 2003), while white decorations (sun-bleached snail shells and bones) are amassed in great number further away from one or both bower entrances (Fig. 1a,b). The number of specific decorations of the former type has been shown to predict mating success (Madden 2003), while the more common white decorations plausibly serve different functions, i.e. signalling bower position from a distance (Borgia 1995a). For this reason, we counted the number of decorations displayed at bowers separately for outer white items and inner bower decorations. Decorations were counted manually at the end of each field season by two independent observers, and the average value for each bower was used for analysis.

*Display ethogram*

Most display elements we identified were described in previous published accounts on this species (Marshall 1954; Warham 1962; Frith and Frith 2004). For each previously described element, all synonyms and the available descriptions are listed in the Supplementary Table S1. As for display elements which were identified here for the first time, these were named after their counterparts in closely-related bowerbird species (Frith and Frith 2004). In relation to previously described display elements, we did not observe what had been defined as ‘Raised-wings’, as we only observed wing flicks as rapid flashing of the wings rather than slow wing lifts as reported in Frith and Frith (2004). Moreover, ‘crest presentation’ only occurred in the ‘forward’ variant in our study population (Fig. 2b), and not with the ‘nape presentation’ described in other studies conducted at different locations (Chartres Towers, see Frith and Frith 2004). Display elements that consist of a short and discrete movement were considered as ‘events’ with no duration (Supplementary Table S1). All display elements were performed either with or without a decoration held in the beak (e.g., Fig. 2e), after decorations were collected from the arena with a stereotypical movement (‘Decoration pick up’). Finally, we refer here to motionless pauses between subsequent display elements as ‘intervals’ (Fig. 1c), though Warham (1962) considered these pauses as a separate display element, defined as ‘upright posture’ (Warham 1962).

**References**

Borgia G (1995a) Complex male display and female choice in the spotted bowerbird: specialized functions for different bower decorations. Anim Behav 49:1291–1301. <https://doi.org/10.1006/anbe.1995.0161>

Chaffer N (1945) The spotted and satin bower-birds, a comparison. Emu 44:161–181

Frith CB, Frith DW (2004) The Bowerbirds: Ptilonorhynchidae. Oxford University Press, Oxford

Griffiths R, Double MC, Orr K, Dawson RJG (1998) A DNA test to sex most birds. Mol Ecol 7:1071–1075. <https://doi.org/10.1046/j.1365-294x.1998.00389.x>

Madden JR (2002) Bower decorations attract females but provoke other male spotted bowerbirds: bower owners resolve this trade-off. Proc R Soc Lond B 269:1347–1351. https://doi.org/10.1098/rspb.2002.1988

Madden JR (2003) Bower decorations are good predictors of mating success in the spotted bowerbird. Behav Ecol Sociobiol 53:269–277. <https://doi.org/10.1007/s00265-003-0583-6>

Madden JR, Lowe TJ, Fuller HV, Coe R, Dasmahapatra K, Amos W, Jury F (2004b) Neighbouring male spotted bowerbirds are not related, but do maraud each other. Anim Behav 68:751–758. https://doi.org/10.1016/j.anbehav.2003.12.006

Marshall AJ (1954) Bowerbirds: their display and breeding cycle. Oxford University Press, Oxford

Warham J (1962) Field notes on Australian Bower-birds and Cat-birds. Emu 62:1–31

**Supplementary Tables**

| Table S1 Ethogram describing the motor display elements identified from video recordings of courtship interactions. All display elements are divided into three categories based on whether they occur on the display arena while the bird is standing (central static), moving towards the bower (central dynamic), or away from it, running in wide circles (peripheral). | | | | |
| --- | --- | --- | --- | --- |
| Display element | **Description** | **Event or duration** | **Category** | **Synonyms** |
| Body ripple | Forward or sideways vigorous movements towards the bower in an undulating and/or jerky fashion; the beak is open, the wings are kept drooping and the tail is alternately fanning during undulations | Duration | Central dynamic | “Highly animated running […] reminiscent of swimming” (Frith and Frith 2004); “body shudder” (Borgia & Presgraves 1998) |
| Chewing | The bird is standing while sticking the tongue out repeatedly in a chewing-like fashion and bobbing the head backwards | Event | Central static | “Kind of vomiting action” (Chaffer 1945) |
| Crest presentation | The bird rigidly bends forward with erected crest | Duration | Central static | Sideways crest-presentation and forward crest-presentation (Warham 1962) |
| Decoration pick up | The bird picks up a decoration with a rapid head movement while bending forward (commonly follows a ‘Crest presentation’) | Event | Central static | “Decorations are repeatedly picked up” (Frith and Frith 2004); “The male may pick up a berry […] and then lay it on the ground” (Marshall 1954) |
| Decoration tossing | Rapid jerking movement of the head that results in the decoration being thrown away from the body | Event | Central static | “Decorations […] are tossed away” (Frith and Frith 2004); “Picking up twigs and stones, and shaking them away from its beak with a sudden sideways flick” (Warham 1962) |
| Head circling | Rhythmic circular movements of the head executed while the bird is bending forward. Usually followed by ‘Head shaking’ | Duration | Central static | “Sideways crest-presentation. […] Jerking his head up and down through a very small arc” (Warham 1962) |
| Head shaking | The bird shakes the head laterally two or three times | Event | Central static | “Males attacking decorations and shaking the head” (Chaffer 1945) |
| Hop | The bird hops upwards or backwards without flapping the wings | Event | Central static | “Jumps upwards and backwards” (Warham 1962) |
| Lateral step | Lateral step in a rigid posture | Event | Central static | “Males hopping sideways in the most awkward-looking manner” (Chaffer 1945) |
| Leap | The bird flaps the wings and leaps upwards or backwards, quickly retrieving its wings to the flanks; tail fans while leaping | Event | Peripheral | ‘The displaying bird leaps upward and/or backward, sometimes simultaneously flicking wings” (Frith and Frith 2004); “Leap upward to its own height” (Warham 1962); “The bird frequently leaps high in the air” (Marshall 1954) |
| Mock attack | The bird rapidly and aggressively rushes towards the bower (or female) and hits the walls with the legs or full body | Duration | Central dynamic | “Body slam” (Borgia 1995b); “dart rapidly forward towards the still and silent female” (Frith and Frith 2004); “males sometimes aggressively attack females” (Borgia and Presgraves 1998; Borgia and Mueller 1992) |
| Nodding | Head quickly bobs upwards with the body in a standing position | Event | Central static | None |
| Rising | Slow ascendant movement in proximity to the bower walls with neck and body stretched up and beak pointing upwards | Duration | Central dynamic | None |
| Sneeze | The bird gives one vigorous jolt, with its body parallel to the ground | Event | Central static | “Lowers body and wings and jerks whole body and head”, “Lowers head and jerks up and down several times” (Warham 1962) |
| Peripheral run 1: Penguin pose | The bird moves with rapid steps with the body in a rigid upright position; beak is often open while wings are folded along the body. Usually accompanied by high-pitched squealing calls | Duration | Peripheral | “The body is erect with neck extended and head held high, tail down, and wings tight against the body, as they move with small rapid steps” (Frith and Frith 2004) |
| Peripheral run 2: Rooster pose | The bird moves with rapid steps with the body stiffly leaning onwards; beak is often open while the wings are kept backwards parallel to the body. Usually accompanied by high-pitched squealing calls | Duration | Peripheral | “Head and tail are held high, wings horizontally away from the body, and the legs moved in a prancing gait” (Frith and Frith 2004) |
| Peripheral run 3: Wings drooping pose | The bird moves with rapid steps with the body stiffly leaning onwards; beak is often open while the wings are drooping from both sides. Usually accompanied by high-pitched squealing calls | Duration | Peripheral | “The male raised his head, stiffened his neck, and opened his beak. His tail was cocked up and his wings drooped loosely from the body” (Warham 1962) |
| Wing flick | The bird flicks one or both wings rapidly | Event | Central static | “Wing flip. A rapid mechanical extension and retraction of the wings” (Borgia and Presgraves 1998) |
| 180-degree turn | The bird turns its back to the bower with the body kept stiffly upwards | Event | Peripheral | None |

| Table S2 Summary of video recording time, bower activity and male activity budgets at bowers in 2018 and 2019 | | |
| --- | --- | --- |
|  | 2018 | 2019 |
| N of bowers monitored | 14 | 14 |
| Daylight hours of camera activity (total; mean ± SD) | 9671.97  690.85 ± 149.05 | 7887.27  563.38 ± 241.76 |
| Hours of bower activity  (total; mean ± SD) | 795.35  56.81 ± 22.85 | 609.70  43.55 ± 19.49 |
| Bower owner attendance (%)  (mean ± SD) | 77.02 ± 10.34 | 55.91 ± 16.14 |
| Subordinate male attendance (%)  (mean ± SD) | 5.74 ± 4.27 | 21.47 ± 11.51 |

| Table S3 Descriptive statistics of attendance and male-male associations between each subordinate male and different established bowers. Data are shown for associations the equal at least 0.5% of the total recording time per bower. | | | | | | | |
| --- | --- | --- | --- | --- | --- | --- | --- |
| Subordinate ID | **Owner ID^1^** | **Time at bower (min)** | **Total recording per subordinate (min)^2^** | **Relative proportion^3^** | **Proportion of attendance at bower^4^** | **Year** | **N bowers attended** |
| 1800 | B07 | 383.23 | 383.23 | 100.00 | 11.55 | 2018 | 1 |
| 1801 | B03 | 241.50 | 1289.73 | 18.72 | 8.06 | 2019 | 2 |
| 1801 | B11 | 1033.23 | 1289.73 | 80.11 | 35.91 | 2019 | 2 |
| 1801 | B11 | 412.63 | 430.80 | 95.78 | 9.12 | 2018 | 1 |
| 1805 | B18 | 163.58 | 163.58 | 100.00 | 7.12 | 2019 | 1 |
| 1805 | B18 | 25.22 | 25.22 | 100.00 | 1.97 | 2018 | 1 |
| 1807 | B03 | 226.87 | 1750.52 | 12.96 | 7.57 | 2019 | 3 |
| 1807 | B07 | 1390.47 | 1750.52 | 79.43 | 28.89 | 2019 | 3 |
| 1807 | B11 | 133.18 | 1750.52 | 7.61 | 4.63 | 2019 | 3 |
| 1807 | B03 | 14.28 | 104.10 | 13.72 | 0.76 | 2018 | 2 |
| 1807 | B07 | 72.38 | 104.10 | 69.53 | 2.18 | 2018 | 2 |
| 1808 | B04 | 457.98 | 825.70 | 55.47 | 14.38 | 2019 | 2 |
| 1808 | B31 | 367.72 | 825.70 | 44.53 | 10.28 | 2019 | 2 |
| 1808 | B04 | 139.25 | 504.80 | 27.59 | 2.83 | 2018 | 2 |
| 1808 | B31 | 365.55 | 504.80 | 72.41 | 6.54 | 2018 | 2 |
| 1809 | B04 | 219.95 | 607.90 | 36.18 | 6.91 | 2019 | 2 |
| 1809 | B52 | 387.95 | 607.90 | 63.82 | 24.48 | 2019 | 2 |
| 1809 | B04 | 55.87 | 57.55 | 97.08 | 1.14 | 2018 | 1 |
| 1810 | B04 | 52.73 | 52.73 | 100.00 | 1.07 | 2018 | 1 |
| 1811 | B04 | 61.77 | 61.77 | 100.00 | 1.26 | 2018 | 1 |
| 1813 | B02 | 679.65 | 686.17 | 99.05 | 15.81 | 2019 | 1 |
| 1815 | B26 | 150.95 | 285.18 | 52.93 | 10.76 | 2019 | 2 |
| 1815 | B50 | 124.65 | 285.18 | 43.71 | 27.60 | 2019 | 2 |
| 1815 | B26 | 35.85 | 145.05 | 24.72 | 2.48 | 2018 | 2 |
| 1815 | B50 | 77.58 | 145.05 | 53.49 | 1.89 | 2018 | 2 |
| 1819 | B02 | 844.25 | 1141.22 | 73.98 | 19.63 | 2019 | 2 |
| 1819 | B03 | 296.97 | 1141.22 | 26.02 | 9.91 | 2019 | 2 |
| 1826 | B47 | 14.10 | 14.10 | 100.00 | 0.56 | 2018 | 1 |
| 1827 | B07 | 322.58 | 334.35 | 96.48 | 6.70 | 2019 | 1 |
| 1833 | B03 | 147.67 | 375.05 | 39.37 | 4.93 | 2019 | 2 |
| 1833 | B29 | 227.38 | 375.05 | 60.63 | 6.69 | 2019 | 2 |
| 1836 | B03 | 129.05 | 447.02 | 28.87 | 4.31 | 2019 | 3 |
| 1836 | B18 | 12.40 | 447.02 | 2.77 | 0.54 | 2019 | 3 |
| 1836 | B29 | 303.92 | 447.02 | 67.99 | 8.94 | 2019 | 3 |
| 1903 | B02 | 48.12 | 93.23 | 51.61 | 1.12 | 2019 | 2 |
| 1903 | B49 | 32.58 | 93.23 | 34.95 | 2.45 | 2019 | 2 |
| 1914 | B30 | 13.78 | 13.78 | 100.00 | 0.69 | 2019 | 1 |
| 1919 | B30 | 20.07 | 20.07 | 100.00 | 1.00 | 2019 | 1 |
| 1923 | B49 | 28.92 | 30.22 | 95.70 | 2.17 | 2019 | 1 |
| 1924 | B49 | 187.05 | 187.05 | 100.00 | 14.04 | 2019 | 1 |
| *^1^Owner ID* refers to the bower owner(s) the subordinate male was filmed with; *^2^Total recording per individual (min)* refers to the total recording time per subordinate male at any bower per year; ^3^*Relative proportion* refers to the ratio between time at bower and total recording per individual; *^4^Proportion of attendance bower* refers to the ratio between time at bower and total recording time for each breeding season at a bower | | | | | | | |

| Table S4 Results of the model for durations of display elements showing for each response variable the effects of display segment and ownership status. Body ripple: n = 2280 observations; 22 males; Crest presentation: n = 2656; 22 males. | | | | | | | |
| --- | --- | --- | --- | --- | --- | --- | --- |
| Variable | Estimate | SE | t | p | lower CI | upper CI |  |
| Body ripple |  |  |  |  |  |  |  |
| Intercept | -0.194 | 0.056 | NA | NA | -0.304 | -0.087 |  |
| Status | -0.044 | 0.074 | -0.602 | 0.549 | -0.185 | 0.107 |  |
| Segment: Return^*,1^ | -0.028 | 0.032 | -0.862 | 0.665 | -0.090 | 0.041 |  |
| Segment: Bower exit^*,1^ | 0.095 | 0.039 | 2.457 | **0.038** | 0.020 | 0.171 |  |
| Crest presentation |  |  |  |  |  |  |  |
| Intercept | -0.405 | 0.046 | NA | NA | -0.497 | -0.317 |  |
| Status | -0.075 | 0.062 | -1.211 | 0.234 | -0.196 | 0.049 |  |
| Segment: Return^*,2^ | 0.003 | 0.024 | 0.118 | 0.992 | -0.044 | 0.053 |  |
| Segment: Bower exit^*,2^ | -0.102 | 0.035 | -2.930 | **0.008** | -0.167 | -0.027 |  |
| ^*^*In bower* is the reference category for Display segment; *Subordinate* is the reference category for Status; SE: standard error; p-value significance level p ≤ 0.050, significant p-values in bold; CI: lower and upper 95% confidence intervals; NA: not reported as having limited interpretation; ^1^ Comparisons with the reference category *In bower*; the comparison between *Return* and *Bower exit* was estimated as -0.123 ± 0.041, t = -2.959, P = 0.009; ^2^ Comparisons with the reference category *In bower*; the comparison between *Return* and *Bower exit* was estimated as 0.105 ± 0.037, t = 2.798, P = 0.014 | | | | | | | |

| Table S5 Results of the three reduced models for proportions of display elements showing for each response variable the effects of display segment and ownership status (n = 66 observations, 22 birds) | | | | | | |
| --- | --- | --- | --- | --- | --- | --- |
| Variable | Estimate | SE | z | p | lower CI | upper CI |
| 1. Peripheral |  |  |  |  |  |  |
| Intercept | -3.003 | 0.157 | NA | NA | -3.350 | -2.710 |
| Segment: Return^*^ | 0.035 | 0.179 | 0.195 | 0.845 | -0.317 | 0.396 |
| Segment: Bower exit^*^ | 1.522 | 0.148 | 10.290 | **<0.001** | 1.254 | 1.819 |
| Ownership status^†^ | -0.158 | 0.145 | -1.089 | 0.276 | -0.447 | 0.134 |
| 2. Central static |  |  |  |  |  |  |
| Intercept | -4.853 | 0.031 | NA | NA | -4.907 | -4.790 |
| Segment: Return^*^ | -0.019 | 0.034 | -0.540 | 0.589 | -0.089 | 0.045 |
| Segment: Bower exit^*^ | -0.325 | 0.037 | -8.731 | **<0.001** | -0.400 | -0.255 |
| Ownership status^†^ | 0.005 | 0.033 | 0.159 | 0.874 | -0.060 | 0.064 |
| 3. Central dynamic |  |  |  |  |  |  |
| Intercept | -1.944 | 0.141 | NA | NA | -2.227 | -1.665 |
| Segment: Return^*^ | 0.105 | 0.128 | 0.821 | 0.412 | -0.133 | 0.355 |
| Segment: Bower exit^*^ | -0.153 | 0.140 | -1.091 | 0.275 | -0.404 | 0.105 |
| Ownership status^†^ | -0.136 | 0.160 | -0.849 | 0.396 | -0.479 | 0.173 |
| ^*^ *In bower* is the reference category for Segment; ^†^*Subordinate* is the reference category for Ownership status; SE: standard error; p-value significance level p ≤ 0.050, significant p-values in bold; CI: lower and upper 95% confidence intervals; NA: not reported as having limited interpretation | | | | | | |

| Table S6 Results of the model for interval duration showing the effect of display segment and ownership status (n = 27705 observations; 22 birds) | | | | | |
| --- | --- | --- | --- | --- | --- |
| Variable | Estimate | SE | df | z | p |
| Interval duration |  |  |  |  |  |
| Count part |  |  |  |  |  |
| Intercept | 3.084 | 0.023 | NA | NA | NA |
| Segment: Return^*^ | -0.016 | 0.019 | 1 | -0.829 | 0.407 |
| Segment: Bower exit^*^ | 0.261 | 0.021 | 1 | 12.283 | **<0.001** |
| Status | -0.008 | 0.029 | 1 | -0.283 | 0.777 |
| Zero-inflation part |  |  |  |  |  |
| Intercept | -1.206 | 0.025 | NA | NA | NA |
| Segment: Return^*^ | 0.016 | 0.036 | 1 | 0.446 | 0.655 |
| Segment: Bower exit^*^ | 0.310 | 0.041 | 1 | 7.473 | **<0.001** |
| Ownership status^†^ | -0.028 | 0.030 | 1 | -0.936 | 0.349 |
| ^*^*In bower* is the reference category for Segment; ^†^*Subordinate* is the reference category for Ownership status; SE: standard error; p-value significance level p ≤ 0.050, significant p-values in bold; NA: not reported as having limited interpretation | | | | | |

| Table S7 Results of the model for decoration use showing for each response variable the effects of display segment and ownership status (n = 69 observations; 23 birds) | | | | | | |
| --- | --- | --- | --- | --- | --- | --- |
| Variable | Estimate | SE | z | p | lower CI | upper CI |
| Intercept | -2.207 | 0.185 | NA | NA | -2.578 | -1.863 |
| Element: Central dynamic^*^ | 1.127 | 0.150 | 7.493 | **<0.001** | 0.828 | 1.426 |
| Element: Central static^*^ | 1.604 | 0.148 | 10.827 | **<0.001** | 1.330 | 1.891 |
| Ownership status^†^ | -0.221 | 0.203 | -1.086 | 0.278 | -0.627 | 0.152 |
| ^*^*Peripheral* is the reference category for Display element; ^†^*Subordinate* is the reference category for Status; SE: standard error; p-value significance level p ≤ 0.050, significant p-values in bold; CI: lower and upper 95% confidence intervals; NA: not reported as having limited interpretation | | | | | | |

| Table S8 Results of the model for attendance rates showing the effect of date, ownership status and year (n = 3120 observations; 1126 days-bower; 16 bowers) | | | | | | |
| --- | --- | --- | --- | --- | --- | --- |
| Variable | Estimate | SE | z | p | Lower CI | Upper CI |
| Intercept | -6.217 | 0.068 | NA | NA | -6.346 | -6.089 |
| Date | -0.019 | 0001 | -14.705 | **<0.001** | -0.021 | -0.017 |
| Ownership status: Unbanded^*^ | -0.528 | 0.076 | -6.969 | **<0.001** | -0.675 | -0.391 |
| Ownership status: Owner^*^ | 1.117 | 0.063 | 17.782 | **<0.001** | 0.999 | 1.233 |
| Year | 0.087 | 0.038 | 2.273 | **0.023** | 0.011 | 0.163 |
| Date:Ownership status(Unbanded) | 0.014 | 0.002 | 8.391 | **<0.001** | 0.011 | 0.017 |
| Date:Ownership status(Owner) | 0.025 | 0.001 | 18.336 | **<0.001** | 0.023 | 0.028 |
| ^*^*Subordinate* is the reference category for Ownership status; SE: standard error; p-value significance level p ≤ 0.050, significant p-values in bold; CI: lower and upper 95% confidence intervals; NA: not reported as having limited interpretation | | | | | | |

**Supplementary figures**

**Fig. S1** Frequencies of attendance at bowers (per total recording time per bower) for 2018 (left) and 2019 (right). Different categories correspond to different classes of birds present at a bower (bower owners, subordinate males, unbanded birds). The category ‘Other’ includes birds with bands or feet not visible, male competitors marauding the bower, females visiting the bower in the absence of the owner or subordinate males.

**Fig. S2** Social relationships between subordinate males and bower owners in 2018 and 2019. Solid squares indicate presence of social interactions between a subordinate male a bower owner, with different shades indicating the magnitude of the association between the two individuals (in terms of percentage of attendance of an individual per total bower recording time). Only attendance frequencies > 0.5 % are shown here.

**Fig. S3** Comparisons between bower owners (N = 13) and subordinate males (N = 18) for the rates of four different behaviours. Rates are calculated as time spent exhibiting a given behaviour per hour of recording at a given bower. (a) Maintaining rate calculated as time spent building the bower structure or rearranging decorations per hour of video recording; (b) Rate of display to other calculated as time spent displaying to a banded or unbanded receiver per hour of video recording; (c) Rate of solo display calculated as time spent displaying solitarily at a bower per hour of video recording; (d) Rate of receiving display calculated as time spent watching a display performed by a banded owner or subordinate male per hour of video recording. P-values show the results of Wilcoxon rank tests comparing behavioural rates between subordinate males and bower owners

**Fig. S4** Kaplan-Meier plots for the model investigating latency to produce a peripheral element after a bower exit. Lines represent survival curves with 95% confidence limits of the two groups (subordinate males and bower owners). X-axis represents time in seconds, y-axis represents the proportion of display bouts not exhibiting a peripheral element. The two ownership status groups do not differ significantly in survival (p = 0.3)

**Fig. S5** Comparison of activity time budgets of subordinate males in 2019. (a) Bar plots depict the total attendance time of subordinate males at 13 different bowers. ‘Subordinate(s) alone’ is the proportion of time in which one or more subordinate males are present at a bower alone, i.e. when the bower owner is not present; ‘With owner: non-male’ is the proportion of time in which one or more subordinate males are present at the bower together with the bower owner, and exhibit behaviours that are not male specific (receiving a display, standing by during a display). ‘With owner: male-specific’ is the proportion of time in which one or more subordinate males are present at the bower together with the bower owner, and exhibit behaviours that are male specific (maintenance and display). (b) Comparison of the rates (min/hour of recording per bower) of two male-specific behaviours exhibited by subordinate males when alone and in the presence of the bower owner. Lines connect observations of the same bower. Box plots show median (black horizontal line) and 25% and 75% quartile

**Fig. S6** Social networks of subordinate and resident male spotted bowerbirds in 2018 and 2019. Red circles represent subordinate males and grey circles represent bower owners; line thickness is proportional to weighted degree. For 2018, bower owners are presented in order of descending mating success (left to right)

**Fig. S7** Histograms of estimates of mean graph strength calculated for 10,000 randomizations of bowerbird social networks in (a) 2018 and (b) 2019, compared to the observed mean graph strength constructed with observed proximity events (vertical red line). Mean graph strengths of the observed networks fall outside the 95% confidence interval of the simulated random networks

**Supplementary videos**

**Video S1** Example of a typical courtship bout in which a courting individual alternates central static and central dynamic display element, with or without a decoration held in the beak. After a bower exit, the (unbanded) receiver positions itself again inside the bower walls (‘In bower’, ‘Bower exit’ and ‘Return’ display segments)

**Video S2** Example of ‘Mock attack’ with body slam into the bower walls. This display element is only found in spotted bowerbirds and is characterized by high intensity levels, which may startle the display receiver (see text)
